# Therapeutic Intervention of COVID-19 by Natural Products: A Population-Specific Survey Directed Approach

**DOI:** 10.3390/molecules26041191

**Published:** 2021-02-23

**Authors:** Christian R. Gomez, Ingrid Espinoza, Fazlay S. Faruque, Md. Mahbub Hasan, Khondaker Miraz Rahman, Larry A. Walker, Ilias Muhammad

**Affiliations:** 1 Department of Pathology, University of Mississippi Medical Center, 2500 N. State St., Jackson, MS 39216, USA; 2 Department of Radiation Oncology, University of Mississippi Medical Center, 2500 N. State St., Jackson, MS 39216, USA; 3 Center for Clinical and Translational Science (CCTS), University of Mississippi School of Pharmacy (UMSOP) & University of Mississippi Medical Center, 2500 N. State St., Jackson, MS 39216, USA; iespinoza@umc.edu; 4 Department of Preventive Medicine, John D. Bower School of Population Health, University of Mississippi Medical Center, 2500 N. State St., Jackson, MS 39216, USA; ffaruque@umc.edu; 5 Institute of Pharmaceutical Science, School of Cancer and Pharmaceutical Sciences, King’s College London, Franklin-Wilkins Building, 150 Stamford Street, London SE1 9NH, UK; Mahbub.hasan@kcl.ac.uk (M.M.H.); k.miraz.rahman@kcl.ac.uk (K.M.R.); 6 National Center for Natural Product Research, Research Institute of Pharmaceutical Sciences, School of Pharmacy, University of Mississippi, Oxford, MS 38677, USA; lwalker@olemiss.edu

**Keywords:** COVID-19, SARS-CoV-2, cytokine storm, natural product, in-silico docking, G6PD, disparities, risk factors, life style variables

## Abstract

To date very few promising leads from natural products (NP) secondary metabolites with antiviral and immunomodulatory properties have been identified for promising/potential intervention for COVID-19. Using in-silico docking studies and genome based various molecular targets, and their in vitro anti-SARS CoV-2 activities against whole cell and/or selected protein targets, we select a few compounds of interest, which can be used as potential leads to counteract effects of uncontrolled innate immune responses, in particular those related to the cytokine storm. A critical factor for prevention and treatment of SARS-CoV-2 infection relates to factors independent of viral infection or host response. They include population-related variables such as concurrent comorbidities and genetic factors critically relevant to COVID-19 health disparities. We discuss population risk factors related to SARS-CoV-2. In addition, we focus on virulence related to glucose-6-phosphate dehydrogenase deficiency (G6PDd), the most common human enzymopathy. Review of data on the response of individuals and communities with high prevalence of G6PDd to NP, prompts us to propose the rationale for a population-specific management approach to rationalize design of therapeutic interventions of SARS-CoV-2 infection, based on use of NP. This strategy may lead to personalized approaches and improve disease-related outcomes.

## 1. Introduction

On 31 December 2019, the World Health Organization (WHO) was notified of a pneumonia of unknown etiology that was spreading among inhabitants of Wuhan City in the Hubei Province of China. Since that time, the causative agent has been identified as severe acute respiratory syndrome coronavirus 2 (SARS-CoV2, previously 2019-nCoV) and its respiratory sequelae referred to as Coronavirus Disease 2019 (COVID-19). As of February, 2021 there have been over 110 million cases of COVID-19 diagnosed globally and nearly 2.5 million deaths attributed to the pandemic. Within the US, over 28 million cases have been diagnosed and more than 500,000 lives have been taken. While reported infections out of China have plateaued, many countries are experiencing exponential spread.

This epidemic raises enormous medical challenges to the scientific community. Researchers work tirelessly to reveal the genetic evolution and the biochemistry of the vital cycle of SARS-CoV-2. Fruits of these efforts could lead to new preventive and therapeutic strategies against COVID-19. So far there is no effective drug treatment to stop the spread of the virus, therefore, to test existing NP as inhibitors of SARS-CoV-2 choosing a molecular target essential to the maturation and production of the virus for screening, such as the main protease 3CLpro (also termed Mpro) which is critical to proteolytic processing of the virus polyproteins, is a good idea. Subsequent in vitro and in vivo experiments for further validation, specifically, the development of novel protocols, based on existing anti-inflammatory and anti-viral NP small molecules (NPSM) represents a valid and alternative therapeutic strategy [1,2]. Concurrently, novel approaches such as in-silico docking studies and genome-based molecular targets should call our attention as methodologies valuable to test existing NP against whole cell and selected molecular determinants. In this review paper, we explore such potential. We focus on the biological basis of virulence as well as the less discussed population-based variables relevant to use of NP, and SARS-CoV-2 virulence. We discuss on the effect of concurrent comorbidities related to SARS-CoV-2 infection. Additionally, we explore the association between use of NPs under inherited glucose-6-phosphate dehydrogenase deficiency (G6PDd)—the most common human enzymopathy—on scope of COVID-19. We do so because the potential of NP to fence infection, boost efficacious immune antiviral response, and protect against respiratory infections such as COVID-19 has been proposed to be decreased in populations with high prevalence of G6PDd, known to be vulnerable to excess oxidative stress, such as that present during SARS-COVID-2 infection [3]. This discussion serves in our review as a rationale for a population-based approach for therapeutic interventions of COVID-19 based on use of NP. In all, we intend to seed discussion on personalized approaches that will result on better deployment of NP for treatment of COVID-19 in underserved populations at high risk.

## 2. Biological and Immunological Determinants of Virulence

The SARS-CoV2 virus belongs to the coronavirus family [4]. The positive-sense single-stranded RNA uses the angiotensin-converting enzyme receptor 2 (ACE2) [4,5,6] for entry. Subsequent distribution via the circulatory system leads to infection to various organs expressing ACE2 such as the heart, liver, kidney, lung, and intestine. Compromise of these organs generates hyperactivation of the immune system, and a systemic response characterized by a cytokine storm that may lead to multi-organ failure, circulatory collapse and death in more severe cases [7,8].

A great majority of the current efforts for treatment of SARS-CoV2 have been focused on controlling the effects of inflammation [9,10]. This is based on the evolutionarily ancient nature of the immune system, and its role on host defense mechanisms against pathogens [11]. By use of pattern recognition receptors (PRRs), pathogen-associated molecular patterns (PAMPs), alarmins, activate cell-based responses to cope infection through mechanisms such as phagocytosis, microbicidal activities and release of inflammatory mediators. A poor outcome of the cytokine storm emerging from SARS-CoV2 infection may result from a pre-exiting propensity to a pro-inflammatory state, driven by a pro-inflammatory milieu. Causes for such a state are thought to arise from both intrinsic defects in innate immune cells and extrinsic factors such as hormones and cytokines [12]. Researchers have focused efforts on understanding the contributing role of the innate immune system primarily because the aberrant inflammatory response, results in immune-mediated damage in patients and leads to more damage than the one inflicted by the virus.

## 3. Approved and Experimental Agents

Currently, there are more than 2680 clinical trials registered at ClinicalTrials.gov which are seeking for effective interventions for SARS-CoV-2 infection (https://www.clinicaltrials.gov/ accessed on 22 February 2021). In October 2020, the FDA approved the antiviral drug VEKLURY (remdesivir) [13]. This is a nucleoside ribonucleic acid (RNA) polymerase inhibitor, with authorized used to treat adult and pediatric patients 12 years of age and older, and weighing at least 40 kg (about 88 pounds) for the treatment of COVID-19 requiring hospitalization (Gilead Sciences, Inc., Foster City, CA, USA). In November 2020, the FDA granted emergency use authorization to two new treatments for COVID-19. Both are based on monoclonal antibodies, bamlanivimab (LY-CoV555) as a single agent (Eli Lilly and Company, Indianapolis, Indiana), and a combination therapy using monoclonal antibodies, casirivimab and imdevimab (Regeneron Pharmaceuticals, Westchester County, NY, USA) [14]. These agents have been approved to treat non-hospitalized adults and children over age 12 with mild to moderate symptoms who have recently tested positive for COVID-19, and those who are at risk for developing severe COVID-19 or are hospitalized because of it. The recommendation for the two therapies includes people over 65, those with obesity or other chronic conditions. Unfortunately, recent results from the ACTIV-3 Phase III trial of Elli Lilly on the investigational monoclonal antibody, LY-CoV555 (bamlanivimab) failed to provide clinical benefits in hospitalized COVID-19 patients, and only the combination therapy from Regeneron is in use in hospitalized patients. However, bamlanivimab is still being evaluated in non-hospitalized patients. Recently, the FDA revised its authorization for high titer COVID-19 convalescent plasma and limited its use only for the treatment of hospitalized patients early in the disease course and to those who have impaired humoral immunity and cannot produce adequate antibody responses [15]. Plasma with low levels of antibodies has not been shown to be helpful in COVID-19 [16]. As numerous experimental agents progress on the pipeline, we expect the emergence of more safe and efficacious treatments to treat COVID-19. Other investigational agents with promising prospects include interferon beta [17,18,19,20,21,22,23], and tocilizumab [24,25]. As of this writing, from the total number of registered COVID-19 related clinical trials, 1993 are actively recruiting or not yet recruiting participants, and we expect new treatments to be available soon.

## 4. Natural Product-Derived Secondary Metabolites as Potential Leads for COVID-19 Therapeutics

Natural products small molecules provide a rich source of novel bioactive compounds and diverse chemical scaffolds compared to the existing class of drugs used for human health. Plants offer a significant and previously untapped resource for antimicrobial and antiviral drug development, with no such currently licensed drugs derived from plant sources despite the comparative abundance of such compounds in other drug settings. Compounds isolated from natural sources, predominantly from plants and microbes have provided many of the therapeutic agents currently on the market. The biological diversity displayed in the natural world reflects an even richer underlying chemical diversity and a vast source of novel molecules with biological activities such as chemical defense or other functions.

Recent advancements in the field of phytochemistry and the technologies related to extraction and pre-fractionation of the extracts, and characterization of the lead compounds from complex mixtures of NP extracts have resulted into availability of highly standardized libraries of NPSM and extracts with assured resourcing of lead extracts or compounds. For example, between 1940 and 2014, out of 175 anti-cancer drugs, 49% approved were either NP) or directly derived from NP [26,27]. In other areas, such as anti-infective agents, the influence of NPSM and their structures is remarkable. A significant number of NP leads/drugs are originally produced by microbes or microbial interaction with host, thereby the areas of NP research have expanded significantly [26,27].

Current approaches of NP antiviral drug discovery efforts have mostly relied on small scale random screening of NP extracts and compounds. The existing repository of NP from plant, marine, and microbial cultures collections offer an unmatched source of NP for new antiviral drug discovery paradigm. In addition to extracts with potent antiviral activity, fractionation of extracts with marginal activity needs to be carried out to ensure that minor critical components should not be missed out in biological screening due to low concentration. Application of pre-fractionation employing sequential solvent extraction and fractionation using C18 cartridges and other high throughput technologies are likely to generate more hits.

## 5. In-Silico Docking Screening as a Strategy for Identification of Natural Products with Potential for Targeting SARS-CoV-2 Infection

To date very few promising leads from NP secondary metabolites have been identified as potential therapeutic interventions for SARS-CoV-2 infection. Thousands of natural compounds have been screened against different SARS-CoV-2 targets [28,29,30]. Through screening studies with different libraries of NP secondary metabolites against different targets, from both SARS-CoV-2 and host using virtual screening, molecular docking and molecular dynamics followed by Absorption, Distribution, Metabolism, and Excretion (ADME) screening have been reported. One study screened 14,011 phytochemicals from Indian medicinal plants deposited to IMPPAT (https://cb.imsc.res.in/imppat/ accessed on 22 February 2021) against host proteases, Transmembrane Serine Protease 2 (TMPRSS2) and cathepsin L, identified qingdainone, edgeworoside C and adlumidine against TMPRSS2 and ararobinol, (+)-oxoturkiyenine, and 3α, 17α-cinchophylline against cathepsin L as inhibitors with high binding affinity, using molecular docking platform AutoDock Vina followed by molecular dynamics simulation [30]. Similarly, 62 alkaloids and 100 terpenoids from local African medicinal plants were tested against 3CLpro (conserved 3-chymotrypsin-like protease; also known as SARS-CoV-2 main protease or Mpro) of SARS-CoV-2 using AutoDock Vina followed by ADME/Tox prediction on SuperPred server (http://lmmd.ecust.edu.cn:8000/ accessed on 22 February 2021) and identified 4 non-toxic alkaloids and terpenoids that bound to the receptor-binding site and catalytic dyad of 3CLpro [31]. Moreover, marine NP libraries (MNP) containing 14,064 compounds were screened by pharmacophore-based virtual screening (Pharmit server; http://pharmit.csb.pitt.edu/ accessed on 22 February 2021), molecular docking (AutoDock and AutoDock Vina on YASARA) and dynamics simulation (YASARA), which revealed 17 high-affinity inhibitors against 3CLpro [32]. Another study utlized Schrödinger Package for pharmacophore-based virtual screening of 409,147 molecules from supernatural product (SNP) database (274,363), Zinc natural database (120,720) and MNP database (14,064) to identify SN00293542, and SN00382835 as inhibitors against 3CLpro [32]. Interestingly, Salvianolic acid A was identified against 3CLpro by screening 32 phytochemicals from 14 cooking spices utilizing molecular docking in AutoDock4.2.6 followed by molecular dynamics in AMBER16 [33]. It is plausible to utilize in silico screening with different combinations of NP secondary metabolites database, different host and/or SARS-CoV-2 receptors and software platforms (open access AutoDock and AutoDock Vina or paid package like Schrödinger) to identify more potential lead candidates from NP against SARS-CoV-2.

## 6. Selected Potential Leads

Using in-silico docking and in vitro anti-viral activity evaluation, we were able to select compounds of interest, namely *bis*-benzyltetrahydroisoquinoline alkaloids (cepharanthine, berbamine, tetrandrine and fangchinoline), triterpenes and saponins (glycyrrhizinic acid and glycyrrhizin), saikosaponins, anthraquinones (hypericin), flavonoids (quercetin and rutin), and polyphenolic compounds. These NPs are abundantly present in various genera of many plant families. Properties that can be exploited as a model or template to carry forward further for the discovery effort of unique therapeutic agents based on NP:

## 7. Dimeric Benzyltetrahydroisoquinoline (bis-btiq) Alkaloids

**Figure 1:**
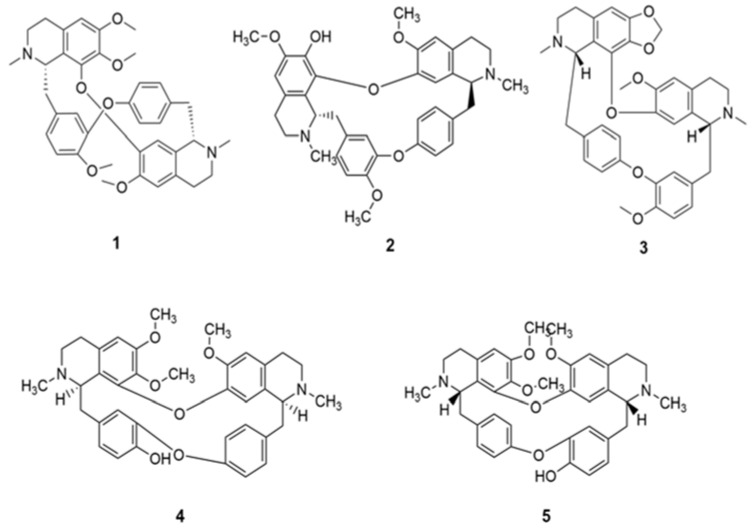
Structures of potential lead *bis*-benzylisoquinoline alkaloids (**1**–**5**).

*Bis*-benzyltetrahydroisoquinoline alkaloids tetrandrine (**1**), fangchinoline (**2**) and cepharanthine (**3**) (Figure 1) significantly inhibited virus-induced cell death at the early stage of infection [34]. Treatment of MRC-5 human lung cells in culture with compounds **1**-**3** dramatically suppressed the replication at low concentrations in tissue culture models of HCoV-OC43 as well as inhibited viral S and N protein expression [34]. Against SARS-CoV-2 and homologous viruses, **3** predominantly inhibits viral entry and replication at low doses; and was recently identified as the most potent coronavirus inhibitor among 2406 clinically approved drug repurposing candidates in a preclinical model [35].

## 8. Berbamine

This *bis*-benzyltetrahydroisoquinoline alkaloid, berbamine (**4**) potently inhibited the infection of various coronaviruses (e.g., SARS-CoV-2 and MERS-CoV), aviviruses (e.g., JEV, ZIKV, and DENV), and enteroviruses (e.g., EV-A71) in host cells, and protected mice from lethal challenge of JEV, as well as inhibited transient receptor potential mucolipins TRPMLs (Ca^2+^ permeable non-selective cation channels in endosomes and lysosomes), which compromised the endolysosomal tracking of viral receptors, such as ACE2 and DPP4. In summary, berbamine, can act as a pan-anti-(+)ss RNA virus drug by inhibiting TPRMLs to prevent viral entry [36].

## 9. Oxyacanthine and Hypericin

A *bis*-benzyltetrahydroisoquinoline alkaloid oxyacanthine (**5**), an analog of CEP, and a dimeric anthraquinone hypericin (**11**) have shown good binding efficacy (via molecular docking values −10.990 and −9.05 kcal/mol, respectively) among others but oxyacanthine was the only NP which made some of necessary interactions with residues in the enzyme (protease) require for target inhibition [37].

## 10. Nelfinavir and Cepharanthine

In a cell culture model (VeroE6/TMPRSS2) of SARS-CoV-2, combination of the human immunodeficiency virus (HIV) protease inhibitor nelfinavir (which binds the SARS-CoV-2 main protease) and **3** (which inhibits viral attachment and entry into cells) showed synergistic effect to limit SARS-CoV-2 proliferation. Combining nelfinavir/cepharanthine enhanced their predicted efficacy to control viral proliferation, to ameliorate both the progression of disease and risk of transmission [38].

## 11. Berbamine, Amlodipine, Loperamide, and Terfenadine

In a preprint paper posted to BioRxiv, researchers (Mount Sinai Hospital) identified one natural alkaloid (berbamine) and three approved synthetic drugs (amlodipine, loperamide, and terfenadine) that could block replication of the novel coronavirus. They then validated these findings in multiple assays using primate vero cells infected with SARS-CoV-2, A549 cells, and in human organoids. “*These compounds were found to potently reduce viral load despite having no impact on viral entry or modulation of the host antiviral response in the absence of virus*,” according to the article [39].

## 12. Glycyrrhizinic Acid (6) and Glycyrrhizin (7)

**Figure 2:**
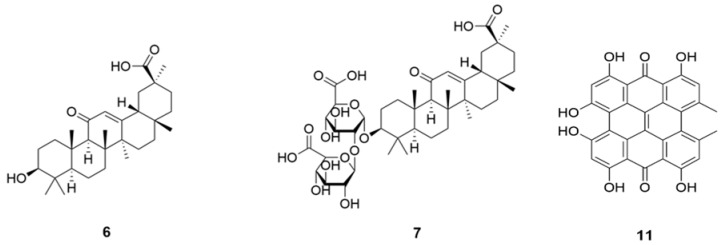
Structures of triterpene and triterpene glycoside (**6** and **7**), and anthraquinone (**11**).

Triterpene glycoside, glycyrrhizin (**7**) (Figure 2) may reduce the severity of an infection with COVID-19 at the two stages of the COVID-19 induced disease process, (1) by blocking the number of entry points and (2) by providing an ACE2 independent anti-inflammatory mechanism. At the membrane level, **7** induces cholesterol-dependent disorganization of lipid rafts which are important for the entry of coronavirus into cells. At the intracellular and circulating levels, **7** can trap the high mobility group box 1 protein and thus blocks the alarmin functions of High Mobility Group-box (HMGB)1 [40,41,42,43].

## 13. Saikosaponins

**Figure 3:**
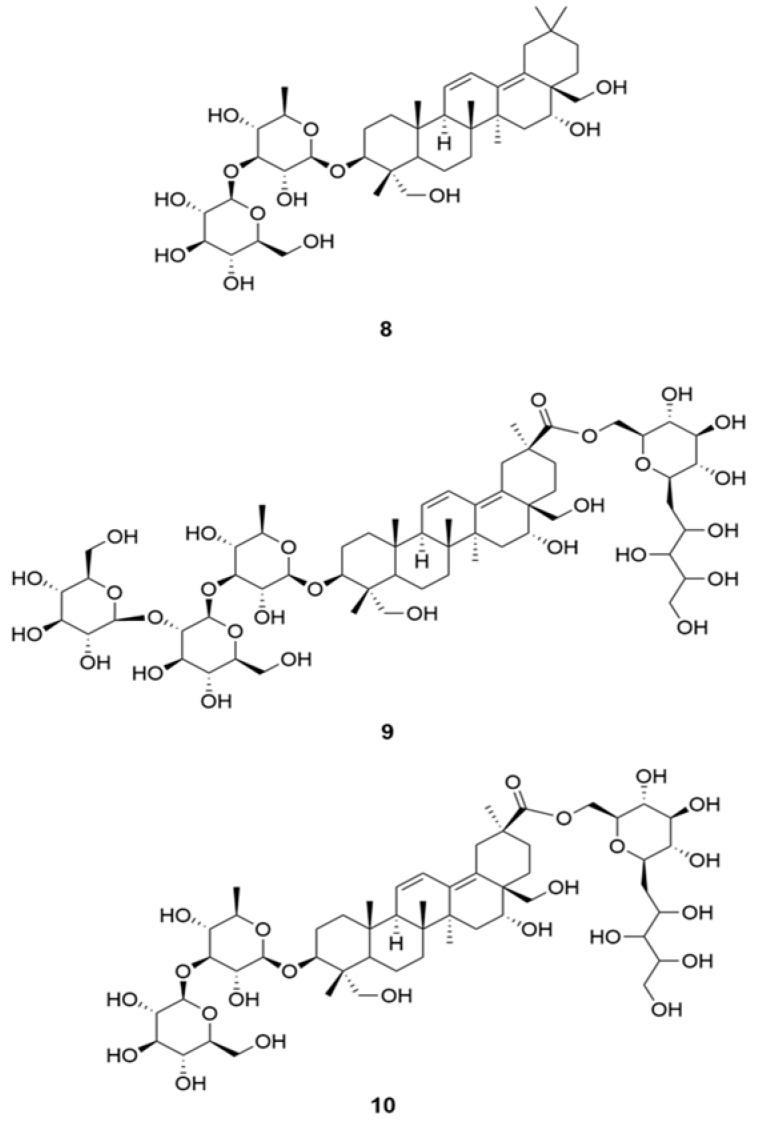
Structures of anti-covid-19 saikosaponins **8**–**10**.

Saikosaponin is a group of oleanane triterpenes reported for multi-functional bioactivity, namely antiviral, antitumor, anti-inflammatory, anticonvulsant, antinephritis, and hepatoprotective activities [44]. They also displayed anti-coronaviral activity by interfering the early stage of viral replication, as well as absorption and penetration of the virus. The potency of different saikosaponins against different sets of SARS-CoV-2 binding protein via computational molecular docking simulations were evaluated [45]. Docking studies were carried out on a Glide module of Schrodinger Maestro 2018-1MM Share Version on NSP15 (PDB ID: 6W01) and Prefusion 2019-nCoV spike glycoprotein (PDB ID: 6VSB) from SARS-CoV-2 [46]. Saikosaponins U (**9**) and V (**10**) (Figure 3) showed the best affinity towards both the proteins, based on binding energy and interaction data, suggesting them to be molecule of interest as they mark the desire interaction with NSP15, which is responsible for 2019-nCoV spike glycoprotein and the replication of RNA, which manage the connection with ACE2 [47]. Additional results indicate that saikosaponin B2 (**8**) has potent anticoronaviral activity and that its mode of action possibly involves interference in the early stage of viral replication, such as absorption and penetration of the virus [44,45,46].

## 14. Flavonoids and Polyphenolic Compounds

The most abundant natural phenolic compounds found in plants, fruit, and vegetables are flavonoids, especially in their glycosylated forms, display a wide array of physiological activities, which makes them interesting to investigate for numerous biological activities, including neuroprotective, antioxidant, antibacterial, and antiviral activities. Due to lack of systemic toxicity, flavonoids and their derivatives may represent unique target compounds to be tested in clinical trials to enrich the drug arsenal against coronavirus infections as well as adjuvant therapy.

Numerous flavonoids were found to have antiviral effects against SARS-and MERS-CoV by mainly inhibiting the enzymes 3CLpro and papain-like protease (PLpro) [48]. However, there are studies focused on flavonoids, polyphenolic compounds, which are proven to be effective against human CoVs. The notable compounds are quercetin (**12**), herbacetin, and isobavachalcone as the most promising flavonoids with anti-CoV potential [48].

**Figure 4:**
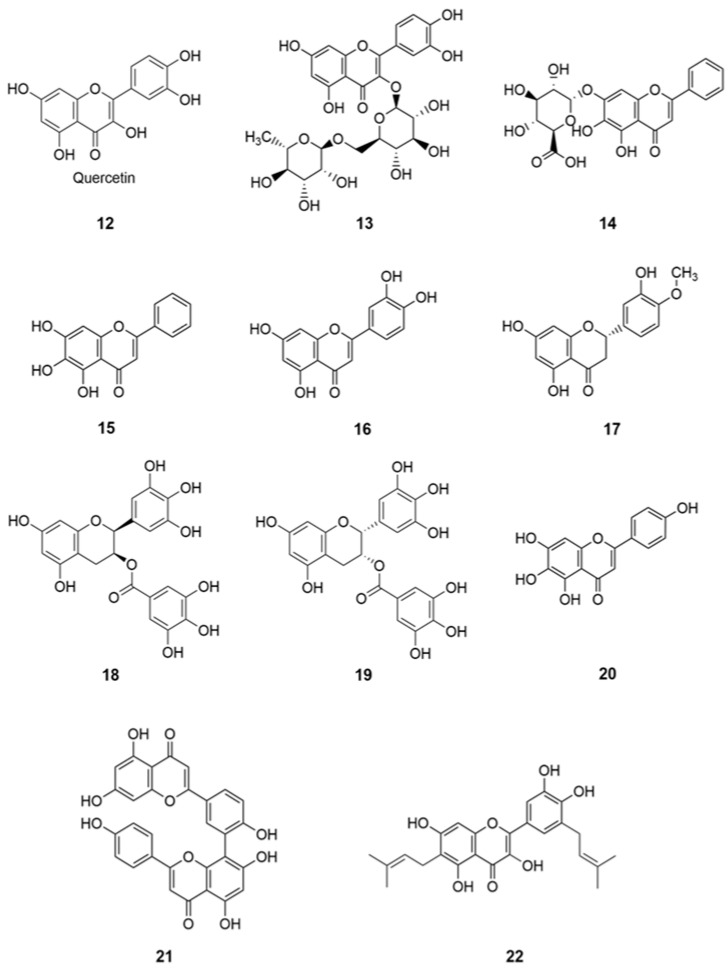
Structures of potential lead flavonoids and phenolic compounds **12**–**22**.

In a recent review, using various in silico and in vitro studies on antioxidative flavonoids, as an alternative or additional therapeutic/preventive option, have interfered with various stages of coronavirus (SARS-CoV-2) entry and replication cycle [1]. The capacity of well- known flavonoids with antioxidant and antimicrobial functions, namely quercetin (**12**), rutin (**13**), baicalin (**14**), baicalein (**15**), luteolin (**16**), hesperetin (**17**), gallocatechin gallate (**18**), epigallocatechin gallate (**19**), scutellarein (**20**), amentoflavone (**21**), and papyriflavonol A (**22**) (Figure 4) inhibited key proteins involved in coronavirus infective cycle, such as PLpro, 3CLpro, and NTPase/helicase [1]. Molecular docking studies using AutoDock Vina revealed Quercetin-3-*O*-rhamnoside showed the highest binding affinity (−9.7 kcal/mol). Docking studies also showed that glycosylated flavonoids are good inhibitors for the SARS-CoV-2 protease [2].

Moreover, two flavonoids, baicalin (**14**) and baicalein (**15**), have recently been identified as novel NP in vitro inhibitors of 3CL protease [49]. These flavonoids could be potential anti-COVID-19 agents [50]. In another study, rutin was identified as the most potential compound based on detailed evaluation and refinement, suggesting the compound might be active against the SARS-CoV-2 3CLpro cysteine protease [51]. In addition, rutin (**13**) has been proved to be active against the flu viruses, and rutin tablets have been used in clinic for many years in China. Therefore, rutin may be a potential inhibitor against SARS-CoV-2 3CLpro [51]. Additional results indicated that the rutin (quercetin-3-*O*-rutinoside) is a potential drug to inhibit the function of chymotrypsin-like protease (3CLpro) of Coronavirus [2].

## 15. NPs for COVID-19 Infection: Specific Demographic Considerations

When efficacy, safety in human, and the long-term effectiveness of traditional approaches to treat COVID-19 remain as open questions, yet NP, due to their effects on acute respiratory infection, generally acceptable toxicity, amenability for oral formulation, and ease of scalability for manufacture make ideal candidates for prophylactic and therapeutic purposes [52].

Studies of applicability of NP to large populations and specifically to high risk populations are very limited [52]. While we have recognized NP’s antiviral and antinflammatory properties, we have not addressed the risk of adverse events. Undoubtedly, this is a matter of concern that will come along with the deployment of NP to the population at large. When we face a global challenge of unprecedented characteristics, exploring safely and efficacy of NP for COVID-19 becomes paramount and highly relevant to those planning practical strategies to contain the pandemic.

In the next sections, we refer to population-specific risk factors and comorbidities related to disparities in response to COVID-19. Additionally, we focus on G6PDd, a prevalent human enzymopathy related to vulnerability to excess oxidative stress, such as that present during SARS-COVID-2 infection. Analysis of demographic and co-morbidity data on COVID-19 and G6PDd obtained from our own medical center provides real world information relevant to a population with cumulative risk factors for disease. This information may be helpful to rationalize selection of NP leads to prevent and treat COVID-19 because, as we will see, population factors such as G6PDd predispose to adverse events after use of NP with antiviral and anti-inflammatory properties. The discussion, therefore is key to design personalized approaches based on use of NP.

## 16. Population-Specific Risk Factors and Comorbidities Leading to Disparities in Response to COVID-19 Infection

COVID-19 has differential impacts on population health. Disparities became obvious due to differences in healthcare resources, underlying health conditions, therapeutic choices and financial capabilities among different populations. Relating COVID-19 with Social Determinants of Health (SDOH), a huge surge of literature emerged since the realization of this pandemic. A recent literature search for the period of 1 January 2020 to 30 December 2020 for the impacts of SDOH on COVID-19 resulted in 8417 unique articles. Some of these studies elaborately discuss the association between SDOH and COVID-19 in the context of population health [53,54,55,56,57]. SDOH related differential consequences of COVID-19 can be due to differential exposure and differential susceptibility to this virus [53,54,58]. Studies show higher burden of COVID-19 on communities with greater social vulnerabilities, particularly with economic inability [59,60].

COVID-19 and population health are often linked by the comorbidities among certain populations. Studies show that patient populations with comorbidities suffer much worse outcomes than patients with no comorbidities [61,62,63,64,65,66,67,68,69,70,71]. Most common comorbidities among COVID-19 patients are hypertension and diabetes [72,73,74,75,76,77]. On the other hand, the prevalence of hypertension and diabetes are much higher in specific populations, such as African Americans (AA). As a matter of fact, most of the underlying diseases of COVID-19 patients are disproportionately prevalent among certain populations [69,78,79,80,81,82,83,84,85,86,87]. Hence, population aspects are crucial in determining the risk factors for COVID-19 infection and outcomes. These comorbidities of COVID-19 are tied with genetics and lifestyles of certain populations.

## 17. Glucose-6-Phosphate Dehydrogenase Deficiency as a Population-Specific Risk Factor for Adverse Outcomes to SARS-CoV-2 Infection

As described in the previous section there is a list of understudied population factors determining virulence and outcomes following SARS-CoV-2 infection, we want to propose that those factors also determine adverse effects to treatment with NP. Let take as example, the potential of G6PDd as a contributing risk factor for adverse outcomes.

Glucose-6-phosphate dehydrogenase is a cytoplasmic enzyme that catalyzes the production of nicotinamide adenine dinucleotide phosphate (NADPH), which is necessary for maintenance of reduced levels of glutathione (GSH) important to protect erythrocytes from oxidative damage and to reduce susceptibility to hemolysis [88]. The most common medical problem associated with G6PDd is hemolytic anemia, which causes fatigue, shortness of breath, and rapid heart rate. Rapid red cell destruction can result in jaundice, and dark urine. G6PD is critical to protecting erythrocytes against oxidative stress, and deficiency may lead to hemolysis in the presence of certain environmental factors such as infections, medications, and foods [89].

Worldwide, more than 400 million people have G6PDd [90,91,92], a trait encoded by a wide variety of mutations on the X-linked gene, and these mutants lead to varying severity of impairment of G6PD enzyme activity. G6PDd is documented to be more prevalent in African, Asian, Latin American, and Mediterranean populations [91,93]. Even in the US, certain populations, such as AA, overseas deployed military population suffer higher rates of G6PDd [89]. It is known that genetic differences in G6PD activity are probably due to the geography-specific genetic composition. Despite high prevalence in endemic regions, there are few reports relevant to disease management in those areas. Hence, COVID-19 patients in populations with higher prevalence of G6PDd should be investigated and treated more carefully.

The role of G6PDd on viral diseases may result from its potential role in oxidative stress metabolism, which also of relevance in the case of COVID-19 disease [94,95]. Since G6PDd results in the redox imbalance in the erythrocytes, due to hemolysis and tissue damage originated from insufficient oxygen transportation, G6PDd might be a risk factor for those infected by SARS-CoV-2 [3,94,96,97].

A study reported that human lung epithelial A549 cells with G6PDd have 12-fold higher viral production in comparison with cells with normal G6PD activity when infected with coronavirus HCov-229E [97]. In addition, the authors found that viral replication in infected G6PDd cells was 3-fold higher than in cells with normal G6PD [97]. Additionally, population-based data suggest that areas with high relevance of G6PDd may be more susceptible to human coronavirus infections. People from specific countries and regions such as Spain and Italy have been particularly affected by the COVID-19 pandemic, with case fatality rates of 12.0% and 14.2%, respectively [98]. Severe G6PDd is more common in the Mediterranean region. In the Italian island of Sardinia alone, G6PDd prevalence ranges from 10% to 15% [99]. Due to the high prevalence of the allelic variants of G6PDd in these regions, G6PDd should be considered among the factors that may account for severity of COVID-19 illness in these countries.

The data suggest a need for G6PD testing in COVID-19 patients, mainly in places and groups of subjects with high incidence of G6PDd. In support to this notion, recent publications, indicate the relevance to explore the association between G6PDd and severity of COVID-19 disease [3,93,100,101]. These reports advocate for screening for G6PDd of patients affected by SARS-Cov-2 as a method to assess patient susceptibility to infection, greater risk for illness, and as a means to guide the recommendation of treatment options.

## 18. Prevalence of G6PDd in a Level 1 Trauma Center Serving a Community Largely Affected by Unequal Burdens of COVID-19

Due to the current knowledge establishing the relevance of G6PDd as a critical therapeutic determinant for effective antimalarial therapy and its potential relevance as a genetic factor determining outcomes related to SARS-CoV-2 infection, we established prevalence of G6PDd at the University of Mississippi Medical Center (UMMC) in Jackson, Mississippi. This Level 1 Trauma Center serves a community largely affected by unequal burdens such as COVID-19 and G6PDd. African American communities such as those served at UMMC, with high prevalence of G6PDd, are acquiring SARS-CoV-2 at a disproportionate rate [102]. Such survey may be helpful to support strategies for rational selection of NP leads for COVID-19 therapy over the basis of population-specific risk factors.

High prevalence of risk factors associated with severity of COVID-19, low socioeconomic status, and other social determinants plague the AA community representing the majority of UMMC patients [102]. Beyond its relevance due to its abundance of social determinants of health disparities, UMMC the only academic medical center in the State of Mississippi represents an ideal setup for development of basic, translational, clinical, epidemiological, and interventional studies to understand, treat, and prevent COVID-19 infection in subjects or communities at risk.

**Figure 5:**
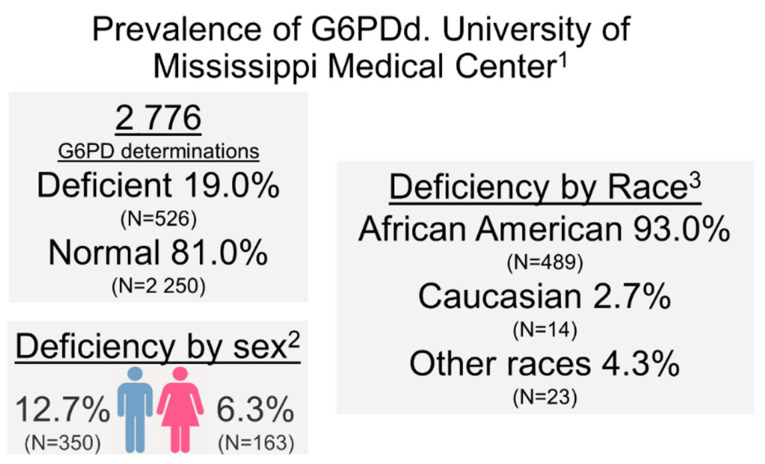
Prevalence of glucose-6-phosphate dehydrogenase deficiency (G6PDd) at the University of Mississippi Medical Center. Superscrips: ^1^ Determinations dated April 2013–September 2020; ^2^ Relative to all determinations 2020; ^3^ Relative to all G6PDd determinations.

We performed a retrospective analysis of de-identified data from the electronic medical records obtained using the UMMC Patient Cohort Explorer application, developed by UMMC’s Center for Informatics and Analytics. Of 2,776 G6PD determinations between April 2013 and September 2020, 526 (19.0%) (Figure 5) were G6PDd, 1785 (64.2%) had normal activity, and 465 (16.8%) had high activity. When stratified by sex, G6PDd prevalence was of 6.3% (N = 163) in females and 12.7% (N = 350) in males. Overall, G6PDd in our study population was largely found among blacks/African Americans, accounting for 93.0% (N = 489) of total G6PDd patients, while other races accounted for very few cases [2.7% for white not Hispanic or Latinos (N = 14), and 4.3% (N = 23) for other races]. It is worthy of note that while the incidence of G6PDd (determined according to enzyme activity) in males is higher than females, the X-linked mutation is carried by heterozygous females, where one normal copy leads to sufficient for normal test activity, but may be in the lower end of the normal range. These “carrier” females can pass the deficient trait to their offspring, more likely to be expressed as true G6PDd in males.

In relation to comorbidities, diagnoses in G6PDd cases were mostly attributed to patients with COVID-19 (5.1%, N = 27) ranked third after HIV (53.4%, N = 281), and anemia (6.3%, N = 33) (Table 1). Consequent with the high incidence of COVID-19 in our region, and the health disparities related to G6PDd, we consider urgent to explore associations in relation to COVID-19 mortality and morbidity on scope of this deficiency. Further investigation is need to elucidate the contributing role of G6PDd to virulence of SARS-CoV-2. Of similar relevance is the identification of traits leading to disparate effects in those with G6PDd. Actionability of such concepts can prioritize personalized prevention and treatment measures for effective treatment.

Despite the limitations related to brief exploration window and a potentially wide range of confounders, the data is valuable. We propose such strategy not only in complex clinical units, but also in underserved areas. Point-of-care assessment of G6PDd must be a companion of rapid SARS-CoV-2 testing. Currently our research group is assessing performance of G6PDd screening modalities using rapid testing. We believe these tools will allow for implementation of community-based therapeutic strategies for treatment of COVID-19 using NP, not only when considering prevalence of G6PDd, but also other relevant risk factors.

## 19. Predisposition to Adverse Events after Use of Natural Products in G6PDd Subjects, a Call for Attention When Treating COVID-19

G6PDd predisposes to acute hemolytic anemia, and can be triggered by products such as herbs, drugs, or infection due to the increase in oxidative stress. Analysis of this connection is necessary due to the proposed link between G6PDd ROS-induced damage and inequities in mortality associated with COVID-19 [3]. The most common demonstrated trigger is fava bean (*Vicia faba*) ingestion [103,104,105]. Others have reported that the topical use of *Lawsonia inermis* (Henna) [106] and consumption of *Acalypha indica* [107,108], as responsible of hemolysis in G6PDd subjects. Similarly, use of food colored with the reddish-orange agent 1-phenylazo-2-naphthol-6-sulphonic acid, which is found in food coloring agent, Orange-RN [109,110] was associated with hemolysis in G6PDd subjects.

As studies suggest an association between G6PDd and adverse effects to pharmacological agents, becomes imperative to study susceptibility, especially if such agents are intended to be used to treat SARS-CoV-2 infection. COVID-19 patients with G6PDd can suffer hemolysis and administering certain so called COVID-19 medications, such as chloroquine or hydroxychloroquine may require extra caution. In the past, an ex vivo study has shown that G6PDd cells are more vulnerable to human coronavirus infection than G6PD-normal cells [97], which should have made researchers interested in examining the association between G6PDd and COVID-19 [111]. Buinitskaya et al. (2020) from their recent review study, reported that G6PDd and SARS-CoV-2 both compromise the anti-inflammatory antioxidant system through the same pathways [112]. In this sense, overproduction of ROS and excess oxidative damage leading to impaired immune responses, over exuberant cytokine storm, and pulmonary dysfunction in response to the COVID-19 won’t be adequately fenced in those with inherited G6PDd. Use of NP aiming the impaired redox status would have the potential to reduce oxidative stress, boost immunity, and reduce the adverse clinical effects of COVID-19 infection in this population. This is an interesting hypothesis worth testing.

The SDOH, which are place-based representations of communities, have known impacts on the COVID-19 transmissibility and outcome disparities. Comorbidities, which are driving the bigger portion of COVID-19 morbidity and mortality, are population specific in general. Age-based population groups show clear distinctions in hospitalization and virulence. The G6PDd can also be interpreted by population as the disorder is genetic and differentially prevalent in certain parts of the world. Thus, the population-related variables contributing to COVID-19 disparities should also be consider as relevant determinants relevant to disease outcome and treatment.

## 20. Conclusions

As emergent therapies to treat COVID-19 are developed, the risk factors that increase the danger of SARS-CoV-2 infection, and the severe side effects that new therapies including NP’s and FDA- approved drugs in combination with NP as adjuvants may cause on these patients, should be considered. Illnesses such as chronic disease and population-specific risk factors should be added into the equation when implementing plans for treatments. Screening and inclusion of these high risk groups in clinical trials will be key to advance in the development of effective COVID-19 treatments not only based on NP but in other therapeutic agents.

## Figures and Tables

**Table 1:** Comorbidities in University of Mississippi medical center patients with G6PDd.

Condition	N (%)
Diseased Related	
Human immunodeficiency virus	281 (53.4)
Anemia	33 (6.3)
COVID-19	27 (5.1)
Sepsis	20 (3.8)
End stage organ disease	20 (3.8)
Autoimmune diseases	17 (3.2)
Therapeutic drug monitoring	17 (3.2)
Cancer	12 (2.3)
Organ transplants	12 (2.3)
Other diseases	63 (12.0)
Non-Disease Related	
Newborns	24 (4.6)

## Data Availability

The data presented in this study are available on request from the corresponding author.

## Abbreviations

Absorption: Distribution: Metabolism: and Excretion	ADME
African Americans	AA
Angiotensin-Converting Enzyme receptor 2	ACE2
3-Chymotrypsin-Like protease	3CLpro
COronaVIrus Disease of 2019	COVID-19
Glucose-6-Phosphate Dehydrogenase deficiency	G6PDd
Marine NP libraries	MNP
Natural Products	NP
Papain-like protease	PLpro
Pathogen-Associated Molecular Patterns	PAMPs
Pattern Recognition Receptors	PRRs
Severe Acute Respiratory Syndrome Coronavirus 2	SARS-CoV-2
Social Determinants of Health	SDOH
University of Mississippi Medical Center	UMMC
